# Role of Calcium Signaling in Prostate Cancer Progression: Effects on Cancer Hallmarks and Bone Metastatic Mechanisms

**DOI:** 10.3390/cancers12051071

**Published:** 2020-04-25

**Authors:** Juan A. Ardura, Luis Álvarez-Carrión, Irene Gutiérrez-Rojas, Verónica Alonso

**Affiliations:** 1 Bone Physiopathology laboratory, Applied Molecular Medicine Institute (IMMA), Universidad San Pablo-CEU, CEU Universities, Campus Monteprincipe, 28925 Alcorcón, Madrid, Spain; juanantonio.ardurarodriguez@ceu.es (J.A.A.); luis.alvarezcarrion@ceu.es (L.Á.-C.); irene.gutierrezrojas@ceu.es (I.G.-R.); 2 Departamento de Ciencias Médicas Básicas, Facultad de Medicina, Universidad San Pablo-CEU, CEU Universities, Campus Monteprincipe, 28925 Alcorcón, Madrid, Spain

**Keywords:** Calcium, prostate cancer, cell signaling, therapies, cancer progression

## Abstract

Advanced prostate cancers that progress to tumor metastases are often considered incurable or difficult to treat. The etiology of prostate cancers is multi-factorial. Among other factors, de-regulation of calcium signals in prostate tumor cells mediates several pathological dysfunctions associated with tumor progression. Calcium plays a relevant role on tumor cell death, proliferation, motility-invasion and tumor metastasis. Calcium controls molecular factors and signaling pathways involved in the development of prostate cancer and its progression. Such factors and pathways include calcium channels and calcium-binding proteins. Nevertheless, the involvement of calcium signaling on prostate cancer predisposition for bone tropism has been relatively unexplored. In this regard, a diversity of mechanisms triggers transient accumulation of intracellular calcium in prostate cancer cells, potentially favoring bone metastases development. New therapies for the treatment of prostate cancer include compounds characterized by potent and specific actions that target calcium channels/transporters or pumps. These novel drugs for prostate cancer treatment encompass calcium-ATPase inhibitors, voltage-gated calcium channel inhibitors, transient receptor potential (TRP) channel regulators or Orai inhibitors. This review details the latest results that have evaluated the relationship between calcium signaling and progression of prostate cancer, as well as potential therapies aiming to modulate calcium signaling in prostate tumor progression.

## 1. Introduction to Prostate Cancer

Genetic and environmental factors contribute to alterations of prostate that may lead to uncontrolled cell growth and prostate tumorigenesis and cancer. Prostate cancer (PCa) is a heterogeneous and multifactorial disease [1]. Heterogeneity is reflected at different levels: (I) at the level of gene expression—not all the cells within a cancer mass express tumor markers to the same extent—(II) at the genetic level—with multiple loci susceptible to be affected [2]; and (III) in patient response to common therapies -tumors show diverse responses to standard chemotherapy treatments- [3].

PCa is asymptomatic in the early stages of the disease. However, in advanced stages PCa can block urine flow from the bladder, invade the adjacent seminal vesicles and metastasize mainly to bone, lung, liver, pleura and adrenals [4,5,6]. Patients with localized PCa can survive long periods of time and a large percent of patients develop skeletal metastases suggesting that bone provides a favorable microenvironment for its localization and progression [7]. Skeletal metastases cause a variety of complications, such as bone pain, fractures, spinal cord compression, and bone marrow suppression severely compromising patients [8,9].

Androgens, male steroid hormones that act through the androgen receptor (AR), are required for prostate development and physiological prostate function [10]. Approximately 80–90% of PCas are dependent on androgens at initial stages. Although serum androgens alone do not promote prostate carcinogenesis, androgen actions and the functional status of AR are important mediators of PCa progression [11]. Therefore, androgen deprivation therapy is the first line treatment for PCa. Therapies based on chemical or surgical castration are directed towards the reduction of serum androgens and inhibition of AR activity [12,13,14]. Over 80% of patients show a positive response to androgen reduction. However, many patients with metastatic PCa will develop castrate resistant PCa after 2–3 years, leading to an increase in mortality [4]. The tumors of these patients are considered to be hormone refractory, in the sense that they progress despite a reduction in serum androgens [11]. Tumor cell growth is sustained in castrate resistant PCa by a diversity of mechanisms including intratumoral or adrenal production of androgens, overexpression of AR or mutated AR forms, ligand-independent activation of AR or stabilization of hyper-responsive AR by chaperones (reviewed in [15]). Neuroendocrine differentiation has also been associated with the progression of PCas to a castrate resistant phenotype and increased mortality [16,17].

PCa is a major cause of morbidity and mortality worldwide. It is the second most frequent cancer in men and the fifth leading cause of cancer death in men. It was estimated that 1.3 million new cases would be diagnosed causing 359,000 associated deaths worldwide in 2018 [18]. Due to the elevated incidence and mortality of PCa, there is an urgent need to determine the key mechanisms of disease development and response to treatments. Identification of biomarkers for disease progression and therapeutic targets is also considered of the utmost importance.

## 2. Role of Calcium Signaling in PCa Progression

Advanced PCa leading to bone metastases involves several phases regulated by mechanisms that are still not fully understood [19]. Calcium signaling has been described to contribute to the development of PCa characteristics and to participate in different phases of tumor progression [20,21,22]. Calcium signals that differ from those of normal cells in amplitude, subcellular localization or kinetics of the signal are characteristic of PCa cells. These differences on calcium signaling affect cell survival, proliferation, differentiation and other processes that contribute to PCa development [23].

Particular calcium-dependent modifications of prostate tumor cell processes rely on altered homeostasis of calcium and calcium-dependent pathways in these cells. Dysregulated calcium homeostasis in PCa depends on changes in the ratio of influx/efflux and storage of calcium compared with non-tumoral cells [23]. Alterations in plasma membrane and endoplasmic reticulum channels, as well as GAP junctions are mainly responsible for the abnormal calcium intracellular levels of PCa cells [24,25]. These alterations cause calcium influx into the cell and mobilization from internal stores by a variety of mechanisms including constitutive calcium entry [26] store-operated calcium entry (SOCE) [27] and store-independent calcium entry (SICE) [28] processes. On the other hand, increased concentrations of intracellular calcium ([Ca2^+^]_i_) due to dysregulated calcium homeostasis and tumorigenic overexpression of calcium-binding proteins result on altered calcium-dependent signaling pathways.

Evasion of apoptosis, self-sufficiency and limitless cell proliferation and promotion of angiogenesis are cancer hallmarks in PCa [29]. In addition, characteristics acquired by prostate tumors that are required for metastatic PCa include; epithelial to mesenchymal transition, pro-migration and invasion features and metastatic colonization of organs [19,29].

A large number of molecules and signaling pathways have been associated with calcium-dependent modulation of processes involved in PCa cancer hallmarks and PCa progression [21,23,30]. Several of them participate in various phases of PCa development. Herein, we briefly review the role of calcium signaling pathways directly involved in different acquired features and stages of PCa.

### 2.1. Evasion of Apoptosis

**Figure 1:**
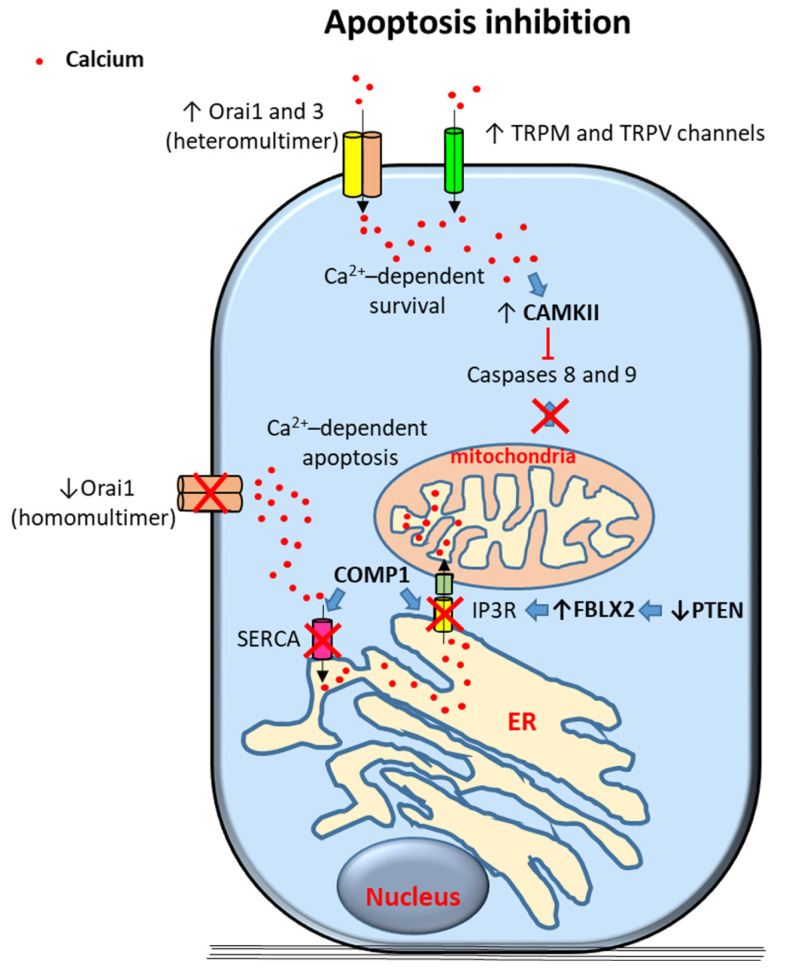
Proposed mechanisms of calcium-dependent apoptosis inhibition in prostate cancer (PCa) cells. Survival signals are induced by calcium entry through transient receptor potential (TRP) TRPM and TRPV channels and Orai 1 and 3 heteromultimers. Elevation of cytoplasmic calcium levels trigger different anti-apoptotic signals including caspase 8 and 9 inhibition by activation of Calcium/Calmodulin-Dependent Kinase II (CAMKII). Alternative mechanisms include inhibition of calcium-dependent mitochodrial apoptosis; excess of intracellular calcium is inhibited by downregulation of Orai homomultimers, of sarco/endoplasmic reticulum calcium ATPase (SERCA) (via cartilage oligomeric matrix protein (COMP) expression) and of IP3R (via COMP1 expression and PTEN (phosphatase and tensin homolog deleted on chromosome 10) channels in PCa cells. Arrows indicate upregulated expression or activity (↑) and downregulated expression or activity (↓). Crosses (X) and ˫ symbol indicate inhibition. Blue filled arrows indicate stimulation. ER: Endoplasmic reticulum. F-box protein XL2: FBXL2.

Calcium has a dual role in cells being able to induce cell survival or triggering apoptosis. For instance, oscillations in [Ca2+]i usually promote cell proliferation and survival whereas sustained cytosolic calcium induces cell apoptosis [31]. Calcium-dependent enhanced cell survival is based on a variety of molecular mechanisms in PCa (Figure 1).

#### 2.1.1. Calcium Channels

A diversity of calcium channels have been involved in promotion of PCa cell survival.

PCa progression has been associated with enhanced Orai3 protein expression [24]. Orai proteins are calcium-channel subunits that form part of the calcium release-activated calcium channels (CRAC) in the SOCE pathway and of the arachidonic acid-regulated calcium (ARC) and leukotriene C4-regulated calcium (LRC) channels in the SICE pathway [24,27,28,32,33]. It has been described that increased levels of Orai3 proteins in PCa cells favors the formation of heteromultimeric channels with Orai1 leading to the formation of SICE channels that resemble ARC channels [24]. OraiI3-Orai1 heteromultimers were proposed to form to the detriment of Orai1 homomultimeric CRAC/SOCE channels [24]. OraiI3-Orai1 heteromultimer have shown to promote cytosolic calcium-dependent proliferation in PCa cells whereas Orai1 homomultimeric channels potentially trigger ER calcium-dependent apoptosis. Given that Orai3-Orai1 heteromultimers could be formed at the expense of homomultimeric Orai1 channels, it has been proposed that Orai3-Orai1 channel predominance confers an oncogenic phenotype of apoptotic resistance and enhanced proliferation in PCa cells [24]. In contrast, another report has described Orai3 downregulation in tumorous versus non-tumorous tissues [34,35]. It was suggested that these contradictory results might be due to selection of different stages of cancer cell differentiation in these studies [36].

A role of members of the transient receptor potential (TRP) calcium ion channel family (reviewed in [37]) on promotion of PCa survival has also been proposed. In this regard, alterations in the expression of TRP Melastatin 2 (TRPM2), TRPM4, TRPM8, TRP Vanilloid 1 (TRPV1) and TRPV6 have been observed in PCa cells [38,39,40,41,42,43,44], some of them related to cancer cell survival. Increased TRPM2 expression in high grade PCa samples has been associated with alterations of autophagy leading to potential consequences on tumor cell survival [38]. Similarly, de novo expression of TRPV6 and translocation of the channel to the plasma membrane via an Orai1/SOCE-mediated mechanism has been shown in PCa cells. TRPV6 translocation would then constitutively increase cytosolic cellular calcium concentrations enhancing PCa cell survival [45]. Regarding TRPM8, experimental data have revealed that this channel modulates cell proliferation, survival, and invasion depending on the cancer cell type and AR requirements. TRPM8, a calcium permeable channel expressed in the endoplasmic reticulum and the plasma membrane that is experimentally activated in response to cooling and menthol has been described to be needed for the survival of AR-dependent LNCaP prostate cancer cells [46]. In contrast, PC-3 are AR-independent PCa cells that express low levels of TRPM8 [46]. In these cells, persistent cytosolic [Ca2+]i due to TRPM8 overexpression by permanent transfection has revealed increased susceptibility to cell apoptosis and decreased proliferation and migration capabilities [40]. Interestingly, TRPM8 has also been associated with inhibition of cell migration via a non-channel function in endothelial cells [47].

Calcium channels mainly located at the endoplasmic reticulum (ER) such as IP3 receptors (IP3Rs) have also been involved in PCa cell survival [48]. These receptors can mediate persistent transport of calcium through ER–mitochondria protein bridges allowing calcium transfer from the ER to mitochondria. This transport can lead to mitochondrial calcium overload and activate the mitochondria-dependent program of cell apoptosis [49]. It has been described that IP3R type3 (IP3R3)-induced apoptosis may be inhibited by F-box protein FBXL2 that targets IP3R3 to proteasome degradation [48]. In turn, activity of FBXL2 has been shown to be inhibited by PTEN (phosphatase and tensin homolog deleted on chromosome 10) tumor suppressor gene. Inactivation or mutation of PTEN tumor suppressor gene is common in PCa and is associated with poor prognosis and metastatic disease [50]. Therefore, it has been suggested that PTEN loss of activity in PCa cells leads to FBXL2 overactivation, IP3R3 proteasomal degradation and inhibition of persistent calcium-dependent mitochondrial apoptosis [48]. ER transfer of calcium to the mitochondria has also been involved in increased mitochondrial activity and subsequent enhanced proliferation and cell survival [51]. In this regard, calcium signals in the mitochondria can be interpreted differentially depending on its spatiotemporal features; intermittent and low calcium levels seem to stimulate metabolism and pro-survival signaling whereas mitochondrial calcium continuous overload results in apoptosis [52].

Several TRP channels are expressed and functional in the ER membrane [53]. Presence of TRP channels, such as TRPM8, has been described in the ER of androgen-sensitive LNCaP cells compared to preferential plasma membrane localization (although comparatively expressed at lower levels) in androgen-insensitive PC-3 cells [46]. TRPM8 localization at the ER membrane has been associated with release of calcium from intracellular stores to the cytoplasm leading to increased survival in AR-dependent LNCaP PCa cells [46].

Cartilage oligomeric matrix protein (COMP) has been shown to be expressed in PCa specimens related to increased growth and recurrence. In vitro, COMP has been associated with inhibition of calcium release from the ER in DU145 cells [54]. In this regard, it has been observed that sarco/endoplasmic reticulum calcium ATPase (SERCA) is inhibited by COMP, thereby blocking ER calcium uptake [54]. Similarly, SOC entry into the ER was also decreased by COMP and thus ER calcium store refilling was diminished [54]. In addition, calcium release from the ER to the cytoplasm through IP3Rs as well as transport of calcium from the ER to mitochondria were hampered by COMP [54]. It has been proposed that these actions altogether result in COMP-dependent decrease of PCa cell apoptosis via inhibition of calcium overload of mitochondria [54].

#### 2.1.2. Calcium-Dependent Proteins and Processes

Several calcium-binding proteins, when bound to calcium, interact with other protein targets to regulate a diversity of cellular functions. Therefore, increased levels of cytosolic calcium caused by overexpression of calcium channels might overactivate calcium-binding proteins—also often overexpressed in PCa—thus acting on processes involved in tumor cell progression.

Calcium/Calmodulin-Dependent Kinase II (CAMKII), among other proteins, seems to play an important role in PCa cell ability to escape apoptosis after androgen deprivation and facilitates the progression of PCa cells to an androgen-independent state. Promotion of PCa cell survival by this kinase is mediated by inhibiting pro-apoptotic triggers caspase-7 and caspase-8 [55].

### 2.2. Self-Sufficiency in Cell Proliferation and Insensitivity to Anti-Proliferatives and Cell Differentiation

**Figure 2:**
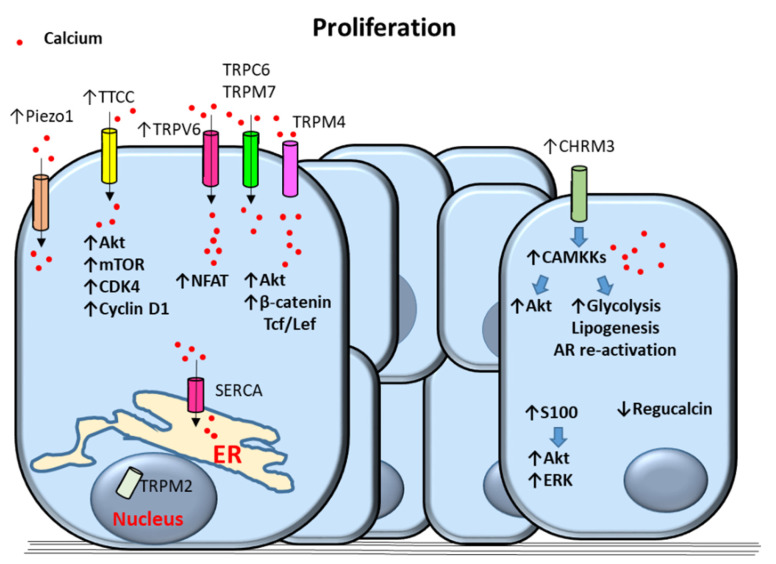
Proposed mechanisms of calcium-dependent proliferation in prostate cancer (PCa) cells. Upregulation of T-Type Calcium Channels (TTCC) increases the proliferative signals Akt kinase, mammalian target of rapamycin (mTOR), cyclin-dependent kinase 4 (CDK4) and cyclin D1. Transient receptor potential (TRP)V6 (TRPV6) increase proliferation via calcium-dependent activation of Nuclear factor of activated T-cells (NFAT). TRPM4 induces proliferation through activation of calcium-dependent Akt and catenin/Tcf/Lef signaling. Piezo1, TRPC6 and TRPM7 contribute to increased calcium cytosolic levels. Nuclear localization of TRPM2 as well as sarco/endoplasmic reticulum calcium ATPase (SERCA) also promote PCa cell proliferation. Upregulation of muscarinic acetylcholine receptor M3 (CHRM3) induces Akt, glycolysis, lipogenesis, and androgen receptor (AR) re-activation via activation of Calcium/Calmodulin-Dependent Kinase Kinase (CAMKK) causing cell proliferation. Proliferation is also triggered by overactivation of Akt and Extracellular-regulated (ERK) kinases by S100 proteins and by downregulation of regucalcin. Arrows indicate upregulated expression or activity (↑) and downregulated expression or activity (↓). Blue filled arrows indicate stimulation. ER: Endoplasmic reticulum.

Androgens play a key role in PCa progression [11] and calcium signaling has been involved in androgen receptor actions on PCa cell proliferation [16]. Furthermore, androgen-dependent increases of [Ca2+]i levels have previously been shown in LNCaP PCa cells [56]. Androgen-independent prostate tumor cell lines express multiple channels that are capable of elevating [Ca2+]i as well [57]. Mechanisms involved in calcium-dependent proliferation of prostate cancer cells are shown in Figure 2.

#### 2.2.1. Calcium Channels

Induction of cell proliferation by physiological stimuli, including epidermal growth factor, serum and androgens, has been described to be controlled by SERCA in LNCaP cells [58]. This study has revealed that the expression of SERCA correlates with PCa cell proliferation and ER intraluminal calcium levels [58].

Overexpression of voltage-operated calcium channel T-type calcium channels (TTCCs) has also been observed in PCa with androgen receptor mutations [59]. Moreover, it has been shown that pharmacological or silencing inhibition of TTCCs causes a decrease in PCa cell proliferation and survival [59]. Based on these observations, it has been proposed that TTCCs control the proliferation of androgen-receptor negative PCa cells [59]. It has also been suggested that an androgen refractory state in which androgen receptor signaling is disrupted causes overexpression of TTCCs and increased cytosolic calcium in PCa cells [16]. In this regard, TTCCs upregulation was associated with tumor progression and the acquisition of neuroendocrine morphological and biochemical changes by PCa cells [16,60].

It has been suggested that TRPV6 expression is upregulated by androgen receptors in a ligand-independent manner in LNCaP prostate tumor cells [61]. Overexpressed TRPV6 channels were described to be constitutively open and act as key mediators of calcium uptake and increased cytosolic calcium in this report. TRPV6-mediated calcium entry was associated with activation of NFAT transcription factor-mediated signaling pathways subsequently promoting cell proliferation [61]. TRP canonical 6 (TRPC6) channels have been proposed as mediators of hepatocyte growth factor (HGF) effects on calcium entry in PCa cells [62]. TRPC6-mediated increase of cytosolic calcium triggered by HGF was shown to induce PCa cell proliferation [62]. In addition, overexpression of TRPM4 in PCa PC3 cells has been associated with increased cell proliferation via activation of β-catenin and phosphorylation of Akt signaling [63]. Both β-catenin and Akt signaling pathways have previously been related to PCa cell proliferation [64]. TRPM4 has been shown to regulate cytosolic calcium concentrations through changes in membrane potential and in calcium electrochemical driving force [65]. In PC3 cells TRPM4 levels positively correlated with enhanced proliferation, Akt activation, protein expression and nuclear localization of β-catenin and transcription of β-catenin target genes dependent on binding with Tcf/Lef transcription factors [63]. In this regard, it has been shown that TRPM4 promotes calcium influx associated with calcium/calmodulin-dependent activation of Akt kinase leading to PC-3 cell proliferation [63]. Similarly, TRPM2 has been associated with PC-3 and DU-145 cell proliferation [66]. Localization of TRPM2 into the cell nuclei has been described to induce cell proliferation in PC-3 and DU-145 cells by an unknown mechanism [66].

Piezo type mechanosensitive ion channel component 1 (Piezo1) is a nonselective cationic mechanosensitive channel able to induce calcium influx in cells [67] that has been described to be overexpressed in PCa cell lines and tissues [68]. Upregulation of Piezo1 has been associated with increased cytosolic [Ca2+]i, phosphorylation of Akt kinase and mammalian target of rapamycin (mTOR), activation of cyclin dependent kinase 4 (CDK4) and cyclin D1 and cell proliferation/survival in DU145 PCa cells [68].

Increased extracellular calcium ([Ca2+]o) levels have also been proposed as modulators of PCa cell proliferation via activation of calcium channels [69]. In particular, increased calcium/magnesium ratios overactivate TRPM7 channels leading to enhanced calcium entry and promotion of DU145 and PC3 PCa cell proliferation [69]. Moreover, increased serum ratios of calcium/magnesium have been observed in PCa patients compared with patients without any cancer [69]. These observations suggest a potential mechanism of PCa progression based on increased [Ca2+]o concentrations that boost cytosolic [Ca2+]i levels. Moreover, increased [Ca2+]o has been described to induce PC-3 proliferation by a mechanism associated with SOC entry and dependent on CasR and TRPC6 expression [70].

#### 2.2.2. Calcium-Dependent Proteins and Processes

Increased cytosolic [Ca2+]i levels switch calcium/calmodulin-dependent kinases from a basal inactive state of auto-inhibition to an active state [71]. Moreover, it has also been described that the androgen receptor is recruited to the calcium/calmodulin-dependent protein kinase kinase 2 (CAMKK2) promoter in both androgen-dependent and castrate-resistant PCa cell lines [72]. Overexpression of the calcium-dependent CAMKK2 protein has previously been described in PCas and cell lines [72]. Therefore, androgen receptor signaling may promote CAMKK2 signaling by both increasing calcium intracellular levels and inducing CAMKK2 protein upregulation.

Previous reports have described that inhibition of CAMKK2 reduces glucose uptake and produces less lactate and citrate suggesting a reduction in aerobic glycolysis. Furthermore, CAMKK2 inhibition showed decreased anabolism from glucose to citrate, ribose and amino acids [72]. Other reports described androgen-dependent CAMKK2 promotion of the glucose transporter GLUT12 trafficking to the plasma membrane [73]. Altogether, these results support the role of CAMKK2 as a mediator of androgen receptor-fueling of PCa metabolism and biosynthesis [72]. In addition, CAMKK2 overexpression has been described to increase the lipogenic enzymes acetyl-CoA carboxylase and fatty acid synthase, thus promoting PCa cell growth by a mechanism dependent on de novo lipogenesis [74]. Furthermore, CAMKK2 upregulation and over-stimulation has been proposed as a mechanism that re-activates androgen receptor signaling in castrate-resistant PCa [75]. A feedback loop in which CAMKK2 is induced by the androgen receptor to maintain this receptor activity and trigger tumor cell proliferation has been proposed during PCa progression [76].

In addition, muscarinic acetylcholine receptor M3 (CHRM3) has been described to be highly upregulated in castration-resistant C4-2B and PC-3 cells and moderately upregulated in the androgen-dependent cell line LNCaP [77]. Activation of CHRM3 revealed to cause castration-resistance growth in LNCaP cells through CAMKK–induced activation of Akt kinase [77].

Estrogens have been shown to induce calcium signaling in LNCaP cells [78] although whether these hormones trigger similar CAMPKK2 actions on PCa cells remains elusive.

Increased and persistent [Ca2+]i stimulates androgen receptor breakdown by the protease calpain that forms a complex with calmodulin in LNCaP cells [79]. The resulting androgen fragments have been associated with tumor cell growth arrest [79]. Moreover, persistent [Ca2+]i has been associated with downregulation of androgen receptor expression in LNCaP cells [80]. These results suggest that PCa cell proliferation could be decreased by constant [Ca2+]i via modulation of androgen receptor levels.

Regucalcin expression has been shown to be decreased in human PCas and downregulated in LNCaP PCa cells by an androgen-dependent pathway [81]. Regucalcin is a calcium-binding protein that regulates [Ca2+]i homeostasis by enhancing calcium pumping activity in the plasma membrane, ER and mitochondria of many cell types [82]. Regucalcin was shown to suppress cell proliferation, inhibit expression of oncogenes, and increase the expression of tumor suppressor genes [81]. 

Proteins of the calcium-binding S100 family have been described to be up-regulated in androgen-refractory and metastatic PCa [44]. One of the members of the S100 family of proteins, S100A16, has been involved in promotion of cell proliferation and metastasis via Akt and extracellular signal-regulated (ERK) kinases signaling pathways in human PCa [83].

### 2.3. Angiogenesis

**Figure 3:**
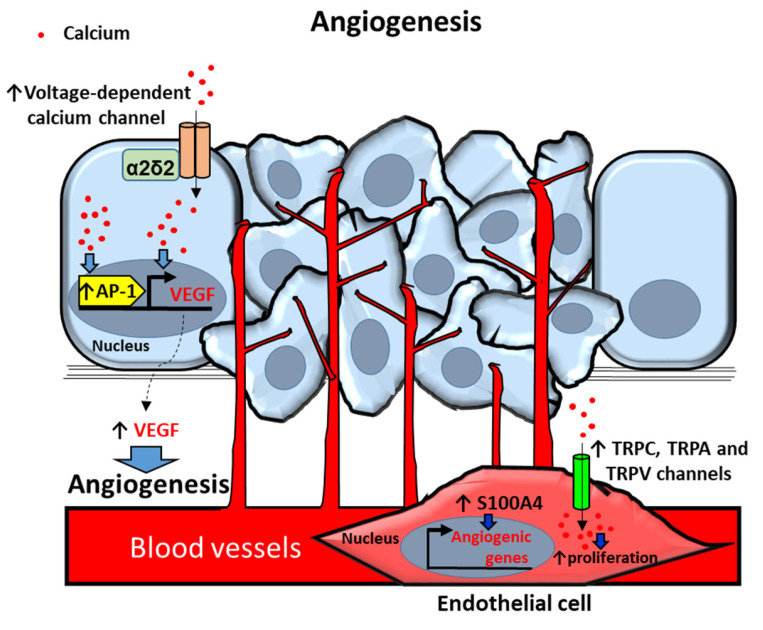
Proposed mechanisms of calcium-dependent angiogenesis in prostate cancer (PCa) and endothelial (EC) cells. PCa cells secrete the angiogenic factor VEGF (vascular endotelial growth factor) by increasing intracellular calcium [Ca2+]i via voltage-dependent calcium channel α2δ2 auxiliary subunit overexpression. [Ca2+]i upregulates VEGF through activation of transcription factor Activator protein 1 (AP-1). ECs in the primary prostate tumor induce angiogenic genes by overexpression of S100 proteins. [Ca2+]i upregulation by Transient receptor potential (TRP) TRPC, TRPA and TRPV channels induces proliferation of ECs in prostate primary tumors. Arrows indicate upregulated expression or activity (↑). Blue filled arrows indicate stimulation.

During PCa progression formation of new blood vessels to supply tumor cell survival and proliferation is required [29]. Constant production of angiogenic factors such as vascular endothelial growth factor (VEGF) frequently occurs in prostate tumor progression (Figure 3).

#### 2.3.1. Calcium Channels

Voltage-dependent calcium channel α2δ2 auxiliary subunit has been reported to be more frequently expressed in PCa tissues compared with non-cancer tissues [84]. Overexpression of this protein has been described to trigger alterations of calcium homeostasis and stimulate angiogenesis via an increased secretion of VEGF in LNCaP nude mice xenografts. These actions have been associated with increased tumorigenesis of LNCaP cells in nude mice and with PCa cell proliferation and tumor development [84].

TRP channels have also been related to angiogenic responses and increased cytoplasmic calcium concentrations. Upregulation of TRPV2 levels has been associated with human prostate tumor-derived endothelial cell proliferation. Moreover, TRPC3 has been identified as an endothelial PCa cell attraction factor whereas TRP Ankyrin1 (TRPA1) has been described to act as a prostate tumor-derived endothelial cell angiogenic factor [85]. These actions were associated with constitutive calcium entry due to basal activation of the three overexpressed TRP channels in endothelial cells and experimentally corroborated with the TRPA1, TRPV2 and TRPC3 agonists Allyl isothiocyanate (AITC), L—Lysophosphatidylcholine(LPC) and 1-Oleoyl-2-acetyl-sn-glycerol (OAG), respectively [85].

#### 2.3.2. Calcium-Dependent Proteins and Processes

It has been described that vasoactive intestinal peptide (VIP) triggers VEGF expression in LNCaP cells. These actions have been attributed to calcium-dependent activation of activator protein-1 (AP-1) response elements in the promoter region of the VEGF gene [86].

In addition, S100A4 calcium-binding protein has been shown to be a key player in development of prostate tumors [87]. It has been described that S100A4 induces capillary formation in endothelial cells in vitro whereas its silencing inhibits angiogenesis and tumor growth in human PCa xenografts of PC3 cells in mice [88]. These effects were associated with alterations in the expression of angiogenesis-related genes in S100A4 knockdown endothelial cells [downregulation of genes related with endothelial migration and microvessel formation; aquaporin-1, fibroblast growth factor 18, resistin, mitogen-activated protein kinase kinase kinase 5 (map3k5), thymus cell antigen, forkhead box O6 (foxo6), heparan sulfate 6-O-sulfotransferase 1 and matrix metalloproteinase 3 (mmp3), and upregulation of anti-angiogenesis genes; cyclin-dependent kinase inhibitor 1A (cdkn1a), thrombospondin 1, and sprouty homolog 4] [88].

### 2.4. Epithelial to Mesenchymal Transition (EMT), Migration and Invasion

**Figure 4:**
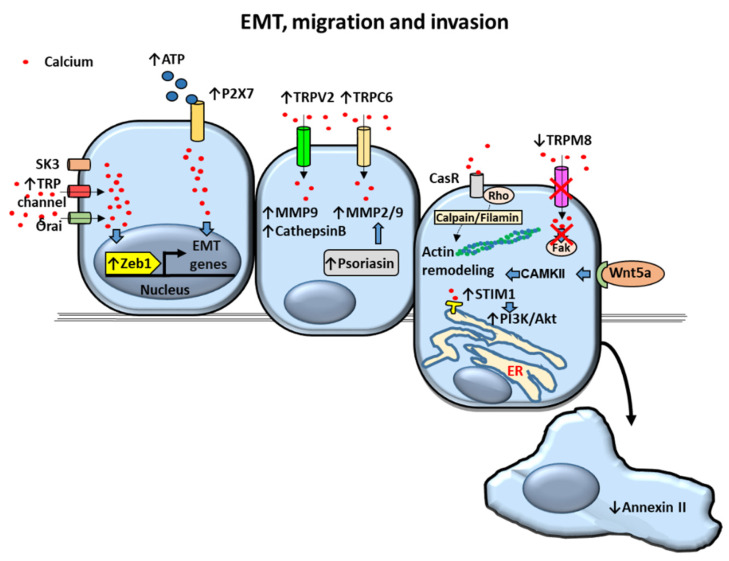
Proposed mechanisms of calcium-dependent Epithelial to Mesenchymal Transition (EMT), migration and invasion in prostate cancer (PCa) cells. Upregulation of intracellular calcium levels dependent on K+ channel (small conductance calcium-activated potassium channel 3) SK3, Transient receptor potential (TRP) and Orai channels overactivate transcription factor Zinc finger E-box-binding homeobox 1 (Zeb1) triggering the expression of *EMT* genes. EMT genes are also activated by ATP-stimulated P2X7 channel. Invasion of PCa cells is mediated by upregulation of metalloproteases (MMPs) and cathepsin B via TRPV2 and TRPC6-dependent increase of cytosolic calcium levels by a constitutive mechanism. MMPs are also increased by psoriasin. Prostate cell migration is promoted by actin remodeling via calcium receptor (CasR)/calpain/filamin and Wnt5a/Calcium/Calmodulin-Dependent Kinase (CAMK)II pathways. Decreased annexin II and increased Stromal-interacting molecule 1 (STIM1)/Akt kinase activation lead to enhanced cell migration as well. Decreased TRPM8 expression decrease in late stages of androgen-insensitive PCA and is associated with increased cell migration. Arrows indicate upregulated expression or activity (↑) and downregulated expression or activity (↓). Crosses (X) indicate inhibition. Blue filled arrows indicate stimulation. ER: Endoplasmic reticulum.

EMT is a process whereby epithelial cells acquire a complete or partial mesenchymal phenotype [89]. EMT promotes a decrease of tumor cell adhesion to the basement membrane and migration of malignant cells from the primary tumor. These processes potentiate prostate tumor cell abilities to migrate to neighbor tissues and to entry into blood or lymphatic vessels [90,91]. EMT allows motility of tumorigenic cells but also contributes to different stages of cancer progression from initiation, primary tumor growth, invasion, dissemination and metastasis to colonization and resistance to therapy [92]. EMT transcriptional program is regulated by transcription factors mainly of the SNAIL, TWIST and Zinc finger E-box-binding homeobox (ZEB) families [92]. Recent findings have reported that calcium entry is required for the upregulation of Zeb1 expression in DU145 and PC-3 PCa cells [93] (Figure 4).

#### 2.4.1. Calcium Channels

It has been shown that calcium-activated K+ channel (small conductance calcium-activated potassium channel 3) SK3 as well as Orai and TRP channels were required for promotion of calcium entry and subsequent Zeb1 expression in these cells [93]. In addition, TRPM7 channel overexpression in DU145 and PC3 was found to increase PCa cell migration mediated through EMT [94,95]. Although promotion of cell migration has been observed to be associated with overexpression of channels such as TRPM7, TRPM4 and TRPM2 [39,94,95,96] the role of calcium on TRPM-mediated cell motility is contradictory. TRPM2 channels induce cytosolic increase of not only calcium but also zinc [96]. Although TRPM2 itself does not directly contribute to calcium entry as a plasma membrane channel, it has been shown that activated TRPM2 induces calcium release from lysosomes contributing to increased cytosolic calcium concentrations in dendritic cells [97]. TRPM2-mediated increase of cytosolic [Ca2+]i has been described to regulate size and number of cell focal adhesions whereas zinc promoted filopodia-cell protrusions required for cell migration- in PC-3 cells [96]. In this regard, migration and motility of PC-3 cells showed to be mediated by TRPM2 in a zinc-dependent rather that calcium-dependent manner [96]. Other reports suggest that promotion of PCa migration by channels is not exclusively due to ion transport. Formation of channel-dependent signaling complexes has been suggested to mediate migration in PCa cells [98]. For example, it has been proposed that the calcium-activated potassium channel BKCa, that is overexpressed in PCa cells, promotes PCa cell migration as well as proliferation [98]. BKCa would act by forming a complex with αvβ3 integrin subsequently increasing phosphorylation of focal adhesion kinase (FAK) in an ion-conducting independent fashion [98].

TRPV2 cationic channel levels are also overexpressed in metastatic PCa compared to primary tumors [99]. It has been shown that introducing TRPV2 into androgen-dependent LNCaP cells enhances cell migration along with expression of invasion markers matrix metalloproteinase (MMP) 9 and cathepsin B. Constitutive activity of TRPV2 showed to mediate the growth and invasive properties of PC3 prostate tumors suggesting that upregulation of this channel is a feature of castration-resistant PCa [99]. Similarly, overexpression of TRPC6 has been observed in PCa samples and different prostate carcinoma cell lines (PC3, DU145, LNCaP and 22Rv1) [100]. It has been described that upregulated levels of TRPC6 promote cell migration and overexpression of metalloproteases MMP2 and MMP9 [100]. Therefore, TRPV2 and TRPC6 role as promoters of proteolytic breakdown of tissue barriers by MMPs to increase PCa cell invasion potential has been proposed [99,100].

TRPM8 expression has been shown to decrease in late stages of androgen-insensitive PCa [101] and TRPM8 overexpression induced by transfection has been associated with reduced PCa cell migration [40,102]. Inhibitory actions of TRPM8 overexpression by transfection on cell migration have been proposed to act through inactivation of the cell migration regulator focal-adhesion kinase in the AR-deficient PC-3 cell line [40]. These actions were associated with persistent cytosolic [Ca2+]i concentrations. In addition, accumulation and activation of TRPM8 channels at the plasma membrane of TRPM8-transfected PC3 cells have been described to be induced by prostate-specific antigen (PSA) related with increased [Ca2+]i and decreased PCa cell migration [102].

#### 2.4.2. Calcium Pumps and Cation Permeable Channels

Plasma membrane Ca^2+^-ATPases (PMCAs) are calcium pumps that use ATP hydrolysis to push calcium from the cytosol into the extracellular milieu. PMCA1 has been identified as a protein that is regulated by the AR in PCa LNCaP cells [103]. Increased secretion of PMCA1 in extracellular vesicles has been associated with inhibition of the AR by the AR antagonist enzalutamide [103]. These results suggest that PMCA1 might have an important role in castrate-resistant PCa and invasion abilities.

Purinergic P2X7 are ligand-gated cation permeable channels activated by ATP that are highly expressed in PCa and PCa cell lines [104,105]. Moreover, extracellular ATP has been described as an important pro-migration and invasion molecule in prostate cancer cells [106]. P2X7 has been involved in ATP-induced enhanced migration and invasion of prostate cancer cells in association with ATP-dependent increase of cytoplasmic [Ca2+]I [105]. The expression of the EMT/invasion-related genes Snail, interleukin-8 (IL-8) and MMP-3 was described to increase whereas the expression of the non-tumor epithelial markers E-cadherin and Claudin-1 was reduced in PC-3M human prostate carcinoma 1E8 and 2B4 cell lines by ATP-activated P2X7 [105].

#### 2.4.3. Calcium-Dependent Proteins and Processes

Stromal-interacting molecule 1 (STIM1), a calcium sensor located in the ER and a component required to induce SOCE, has been shown to be upregulated in PCa [107]. Overexpression of STIM1 has been described to mediate migration and invasion in LNCaP, PC-3 and DU-145 PCa cell lines via activation of the phosphatidylinositol 3-kinase (PI3K)/Akt signaling pathway [107].

Dysregulation of the annexin family of calcium-binding proteins has also been associated with PCa progression [108]. Decreased or absence of annexin II has been shown in Du145 and PC3 PCa cells, respectively. Re-expression of annexin II in these cells inhibited PCa cell migration without affecting cell proliferation or apoptosis [109].

[Ca2+]o has been described to promote the migration of DU145 and PC-3 PCa cell lines (AR-deficient and metastatic) compared to LNCaP PCa cells (AR-positive and less metastatic) [110]. Regarding cell motility, cleavage of filamin A, an actin-binding protein overexpressed in PCa, was shown to be induced by [Ca2+]o [110]. Filamin A cleavage triggered by [Ca2+]o via a calcium-sensing receptor (CasR)-p115RhoGEF-calpain dependent pathway revealed to be essential for promotion of DU145 and PC-3 cell migration [110].

Calcium/calmodulin-dependent protein kinase kinase β (CaMKKβ) has shown to be upregulated in PCa too [111]. Expression and activity of CaMKKβ was described to be increased by androgens leading to phosphorylation of AMP-activated protein kinase (AMPK) [111]. The pathway CaMKKβ/phosphorylated AMPK was shown to induce androgen-mediated migration and invasion of LNCaP and VCaP cells [111].

Wnt5A is another protein that has been shown to be upregulated in PCas [112]. Stimulation of PCa cells with Wnt5A was described to cause [Ca2+]i waves and subsequent activation of CAMKII [112]. CAMKII calcium-dependent activation showed to be indispensable for actin cytoskeleton remodeling and increased motility in PC3 PCa cells [112].

Proteins of the S100 family have also been implicated in tumor cell invasion. The calcium-binding protein psoriasin (S100A7) has been shown to be expressed in PCa specimens and to increase PCa cell survival [113]. However, psoriasin main function was mainly related to increased cell invasiveness abilities through upregulation of matrix metalloproteases in PC-3 cells [113]. Other member of the S100 family of proteins, S100A4, has been reported to be overexpressed in PCa and increase the proliferative and invasive capabilities of PC-3 cells [114]. In this report, enhanced invasion was associated with S100A4-induced transcriptional activation and increased proteolytic activity of the metalloprotease MMP-9 [114].

### 2.5. Homing of PCa to Bone

**Figure 5:**
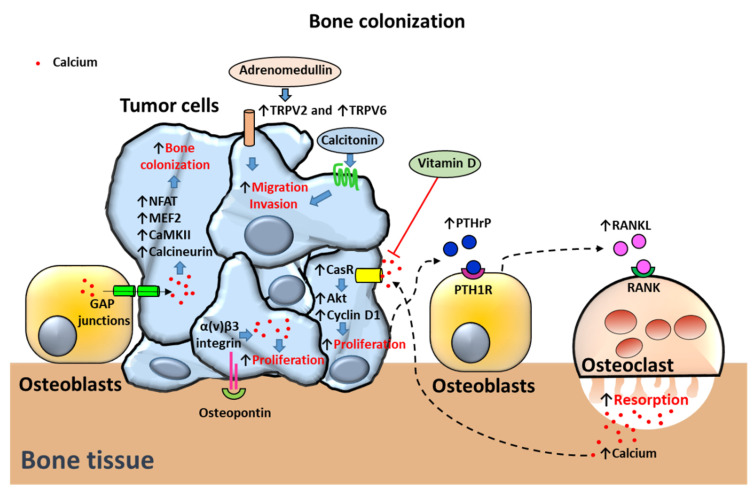
Proposed mechanisms of calcium-dependent bone colonization in prostate cancer (PCa) cells. Migration to bone and invasion mechanisms are induced by Transient receptor potential V2 TRPV2 and TRPV6-dependent upregulation of cytosolic calcium levels in PCa cells. Adrenomedullin translocates TRPV2 to the membrane triggering migration and invasion mechanisms. Calcitonin induces migration and invasion of PCa cells. Bone osteoblasts transfer calcium to tumor cells via GAP junctions. In turn, cytosolic calcium induces bone colonization by overactivation of NFAT and MEF2 transcription factors and calcium-binding proteins CaMKII and calcineurin. Proliferation of PCa cells in bone is triggered by osteopontin activation of α(v)β3 integrin-dependent upregulation of intracellular calcium levels. PCa cells also secrete the bone resorbing peptide parathyroid hormone-related protein (or PTHrP) inducing receptor activator of nuclear factor-κB (RANK) ligand (RANKL) secretion by osteoblasts. RANKL activates RANK receptor in osteoclasts promoting osteoclast-dependent bone resorption and release of calcium. [Ca2+]o activates the calcium receptor (CasR) in PCa cells triggering cell proliferation via Akt and cyclin D1 activation. Vitamin D antagonizes the effects of high extracellular calcium concentrations on CasR. Arrows indicate upregulated expression or activity (↑) and downregulated expression or activity (↓). Crosses (X) and ˫ symbol indicate inhibition. Blue filled arrows indicate stimulation.

Advanced PCa most frequently metastasize to bone, followed in frequency by lung, liver, pleura and adrenals [6,115]. Thus, bone metastasis is a common complication in advanced stages of patients with PCa [115]. It has been hypothesized that tumor cells establish in specific areas of bone such as the endosteal niche, the niche of hematopoietic stem cells and the vascular niche [116]. These niches are complex microenvironments in which bone cells secrete factors and stablish cell-cell interactions that promote cell proliferation and differentiation as well as bone turnover (bone resorption and formation). It has been shown that increasing the number of these niches experimentally also increases the number of primary tumor disseminated cells [117]. Therefore, the PCa high predilection for skeletal metastasis has been attributed to favorable reciprocal interactions between the bone microenvironment and cancer cells [118,119]. Such interactions may be different in nature, including actions of calcium channels, [Ca2+]o, bone soluble factors, bone-tumor cell-cell direct communication and bone matrix proteins (Figure 5).

#### 2.5.1. Calcium Channels

TRPV6 calcium channel overexpression has been associated with development of osteoblastic bone metastasis in addition to promotion of PCa cell survival [45]. PC-3 clones overexpressing TRPV6 have shown to generate osteoblastic lesions compared with control PC3 cells which generate osteolytic lesions when inoculated in the bone marrow of immunodeficient mice [45].

#### 2.5.2. Calcium-Dependent Proteins and Processes

[Ca2+]o: During the process of bone turnover, calcium is the main inorganic component released to the extracellular medium. Normally, physiologic calcium levels are kept within a narrow range of 1.1 to 1.3 mmol/L [120]. However, in active bone resorptive lacunae, [Ca2+]o can reach levels as high as 8 to 40 mmol/L [121,122]. In this regard, [Ca2+]o has been involved in promotion of PCa cell metastasis to bone. [Ca2+]o acts mainly through activation of the heterotrimeric G-protein–coupled receptor CaSR [110]. Effects of [Ca2+]o have been associated with overexpression of calcium-sensing receptor (CasR) and activation of Akt kinase signaling pathway in PC-3 and C4-2B cell lines [7]. Stimulation of CasR and Akt pathways has also been shown to favor metastatic progression in vivo [7]. In vitro, cyclin D1-dependent proliferation and cell attachment of PC-3 cells was enhanced by activation of CasR and Akt, suggesting that [Ca2+]o mediates PCa bone metastasis [7]. Furthermore, the deleterious actions of [Ca2+]o on PCa progression have been associated not only with CasR upregulation but also with overexpression of TRPC6 [70]. Vitamin D has shown to antagonize the effects of high [Ca2+]o concentrations on PCa causing downregulation of both CasR and TRPC6 proteins [70].

It has been observed that elevated [Ca2+]o also stimulates PTHrP secretion in PCa cells [123,124]. PTHrP is a peptide that binds PTH receptor type 1 (PTH1R) in osteoblasts. PTH1R-stimulated osteoblasts secrete the pro-resorption factor RANKL that activates RANK in osteoclasts promoting bone resorption and calcium release [7]. Bone metastases have been associated with activation of the PTHrP-calcium-CaSR axis. It has been proposed that PCa cells cause PTHRP-dependent increase of calcium release from the bone microenvironment. In turn, increased [Ca2+]o levels activate CasR in PCa cells promoting tumor cell proliferation and supporting PCa cell homing to bone [7].

Bone soluble factors: Some reports have shown that bone cells can decrease [Ca2+]i levels while others have described an increase of [Ca2+]i levels in PCa cells [25,118]. This apparent contradiction might be explained due to the existence of dual mechanisms; some would promote high [Ca2+]i levels that sustain processes of prostate tumor bone colonization while others are activated to evade calcium-dependent apoptosis due to overload of mitochondrial calcium. In this regard, bone-metastatic PC3-ML PCa cells have shown downregulated levels of cytoplasmic [Ca2+]i levels upon agonist stimulation via decreased calcium entry when co-cultured with osteoblasts in vitro [118]. A role of the osteoblast microenvironment on reducing apoptosis of PCa cells caused by overload of cytoplasmic [Ca2+]i levels was suggested [118]. In contrast, other studies describe upregulated levels of [Ca2+]i upon stimulation with soluble factors. The role of TRPV2 has been described to mediate adrenomedullin promotion of PC-3 migration, adhesion and invasion abilities. Adrenomedullin, a peptide overexpressed in PCa, was shown to induce TRPV2 translocation to the plasma membrane via a PI3 kinase pathway [125]. In turn, TRPV2 translocation to the plasma membrane caused an increase of the resting cytosolic calcium levels of PC-3 cell line, which induced PCa cell migration, adhesion and invasion [125]. Some studies have linked adrenomedullin and other members of the calcitonin family of peptides including calcitonin itself and calcitonin gene-related peptide to the tropism of PCa to the bone (reviewed in [126]). Although calcitonin exerts hypocalcemic effects by inhibiting bone resorption, it has been described to be highly expressed in malignant prostate tumors and to promote PCa cell proliferation and invasion [126,127].

In addition, soluble factors from bone cells have been related to altered [Ca2+]i levels and increased proliferation in PCa cells [128]. Stimulation of PC-3 cells with conditioned media of pre-osteoblastic MC3T3-E1 and osteocytic MLO-Y4 cells induced an increase in PC-3 cell proliferation. Associated with these actions, osteoblastic and osteocytic conditioned media also caused transient increase in [Ca2+]i accumulation [128].

Bone-tumor cell-cell interactions: Bone colonization has revealed to be mediated through activation of the calcium transducers CaMKII and calcineurin in PCa cells [25]. Moreover, overexpression of transcription factors downstream of calcium and associated with promotion of EMT, migration, angiogenesis and invasion, such as NFAT and MEF2 [129,130] were also observed in PCa bone metastases [25]. It was suggested in these studies that bone colonization requires calcium flows from osteogenic cells to cancer cells via connexin 43-based gap junctions [118]. In this regard, it has been proposed that entry of calcium through calcium channels (Orai for example) mediate initial prostate tumorigenesis whereas bone-cancer cell to cell communication via gap junctions is responsible for bone metastasis after initial tumorigenesis [25].

Bone extracellular matrix proteins: Bone matrix protein fractions have been described to induce rapid fluctuations in cytosolic [Ca2+]i associated with PCa cell proliferation [131]. The non-collagenous matrix proteins osteonectin and osteopontin were able to trigger calcium signals in PCa cells derived from bone (PC-3), but not from lymph-nodes (LNCaP) or brain (DU-145) cells [131]. Effects of osteopontin on calcium signaling were described to be mediated by α(v)β3 integrin in PC-3 cells [131].

## 3. Targeted Calcium Signaling Therapies in PCa

The therapeutic field of PCa has broadened over the last years. These advances coincide with better understanding of the underlying molecular processes of PCa [132]. Aside from calcium-binding proteins, promising new therapies for treatment of PCa include compounds that mainly target various calcium channels and transporters [133]. Table 1 summarizes relevant clinical trials regarding prostate cancer treatment using calcium-targeted therapies.

### 3.1. IP3R Receptor Targets

Unlike in other types of cancers altered IP3R activity has not been extensively described in PCa. However, an unexpected dependency on IP3R-mediated calcium transfer to mitochondria for viability of PCa cells has been found [51]. Treatment with xestospongin B (XeB), a specific IP3R inhibitor, has shown diminished formation of colonies by tumorigenic prostate PC3 cells. Furthermore, XeB-mediated killing and morphological changes including rounding up and shrinkage were observed in PC-3 and DU145 cells. Interestingly, little effect on viability and normal morphology on non-tumorigenic PNT2 prostate line were observed with XeB [51].

On the other hand, the BH4 domain of the anti-apoptotic protein Bcl-2 has been shown to inhibit calcium-mediated apoptosis by inhibiting IP3R-mediated calcium release [134,135]. Interestingly, increased levels of Bcl-2 are required for the progression of prostate cancer cells from an androgen-dependent to an androgen-independent growth stage [136]. Moreover, Bcl-2 upregulation is necessary for androgen-independent prostate cancer cell survival [136]. Therefore, targeting of Bcl-2/IP3R interactions could be a potential therapy in several cancer types including PCa [137]. In this regard, synthetic peptides such as Bcl-2 IP3R Disruptor-2 (BIRD2) are being developed [137]. BIRD2 disrupts Bcl-2-IP3R interplay by binding to the BH4 domain of Bcl-2 and inhibiting Bcl-2 control of IP3R leading to calcium-mediated apoptosis [137]. Whether this approach may be useful has been argued to be dependent on several factor including the levels of Bcl-2 in different stages of cancer cells and their reliance on Bcl-2 for survival [137].

### 3.2. Ca2+ -ATPase Inhibitors

#### 3.2.1. SERCA Inhibitors

Thapsigargin (Tg), a potent inhibitor of ER calcium ATPase (i.e., *ATP2A2* or SERCA 2b) pumps [138] causes an increase in apoptotic death of metastatic castration-resistant PCa cells [139] and anti-proliferative effects after several days of treatment. Furthermore, it has been shown to inhibit the lysosomal degradative autophagy pathway in LNCaP cancer cells [140]. Thapsigargin also inhibits tumor angiogenesis, becoming an ideal agent to annihilate all the cell types present within the cancer microenvironment [141]. One benefit of using Tg compared with most common used chemotherapeutics is its ability to induce apoptosis on both proliferating but also non-proliferating cells. Use of Tg as an antineoplastic agent would require specific targeting towards cancer cells by chemical modification. Coupling to a peptide carrier to produce a water soluble prodrug that targets specifically metastatic accumulations of androgen independent prostate cells would be an option [142]. In this regard, prostate-specific membrane antigen-specific peptides coupled to analogs of thapsigargin (i.e., G202) have been tested. Some of these new analogues have shown a solid correlation between SERCA inhibition and cell death [143] whereas others behave as weak inducers of cell death and barely act as anti-proliferatives [144]. For example, G202 has been described to produce significant regression of a variety of human tumor xenografts in mice [145]. This approach is currently being tested as a clinical trial in patients with advanced solid tumors (ClinicalTrials.gov Identifier: NCT01056029).

#### 3.2.2. PMCA Inhibitors

Recently, PMCA has been identified as putative chemotherapeutic target in advanced stages of PCa [133]. It has been described that resveratrol derivatives may increase [Ca2+]i by inhibiting PMCA and by activating calcium release from the ER. These actions have been associated with decreased PC-3 cell viability [146]. Moreover, compounds based on esterification of resveratrol at the 4’ hydroxyl with 4 carbon acids have shown to enhance [Ca2+]i levels and cause lower PCa cell survival compared to unmodified resveratrol [146]. 

### 3.3. Calcium Channels or Transporters-Targeted Therapies

The expression and/or activity of a large number of calcium channels or transporters are altered in PCas. Compounds or antibodies targeting some of the aforementioned cancer-involved calcium channels/transporters/pumps have been assessed in pre-clinical studies or even in clinical trials [133].

#### 3.3.1. Inhibitors of Voltage-Gated Ca2+ Channels

Neuroendocrine PCa cells derived from LNCaP cells overexpress CaV3.2 T-type voltage-dependent calcium channels (TTCCs) [147]. These channels are also expressed by neuroendocrine cells in PCa tissues obtained from patients after surgery [148]. Recent reports have demonstrated that stimulation of LNCaP cells with bicalutamide—an antiandrogen compound—or hormone-depleted media evoke a significant increase in Ca_v_3.2 protein expression and the appearance of functional T-type Ca^2+^ channels. These channels have been described to induce promotion of chemoresistance to docetaxel, a chemotherapy compound. Regarding these observations, inhibition of T-type calcium channels by sodium butyrate caused a significant reduction in LNCaP survival [149]. Other Cav3.2 channel blockers such as Ni^2+^ or NNC 55-0396 caused a significant reduction in the viability of LNCaP cells exposed to bicalutamide. However, co-treatment with docetaxel and T-type Ca^2+^ channel inhibitors had no further effect on cell viability [16].

Various research studies have found different effects of ghrelin as a treatment for PCa. It has been published that ghrelin inhibits proliferation of human prostate carcinoma cells through T-type calcium channel overexpression [150]. However, no effects of unacetylated ghrelin (UAG) administration on subcutaneous PC3 xenograft growth or metabolic parameters in a mouse model were found, suggesting that UAG is not likely to be an effective treatment for PCa [151]. Recent data has shown limited short-term effects on human PCa xenograft growth by the ghrelin receptor antagonist [D-Lys3]-GHRP-6 [152]. Therefore, further studies are required to elucidate the role of ghrelin and T-type voltage-dependent calcium channels in PCa therapy.

#### 3.3.2. Transient Receptor Potential Channel Inhibitors

Targeting TRP channels has been suggested as a novel therapeutic strategy for PCa [133]. A TRPM8 channel truncated isoform (4TM-TRPM8) has been identified in PCa. Transcription of TRPM8 and 4TM-TRPM8 has been described to be regulated by short truncated TRPM8 isoforms, known as sM8. The suppression of sM8 isoforms by RNA silencers was shown to induce ER and mitochondrial oxidative stress, p21 induction and apoptosis in PCa cells [153]. 

TRPV6 has been described as an oncochannel and several TRPV6 inhibitors have been suggested as potential pharmacological therapies in PCa [133]. A peptidic inhibitor of TRPV6 (SOR-C13) has completed phase I in a clinical trial [154] and has been shown to reduce growth in cell and animal models of PCa [155,156,157].

### 3.4. STIM1 Inhibitors

ML-9, an inhibitor of Akt kinase and STIM1, is emerging as an interesting therapy for PCa. ML-9 induces cell death in PCa cells related to autophagy regulation and enhances the anticancer activity of docetaxel, suggesting its potential application as an adjuvant to existing anticancer chemotherapies [158]. This report suggests to use the chemical structure of ML-9 as a “template” for the synthesis of improved structurally related and more selective compounds to use in cancer treatment.

### 3.5. Purinergic Receptor

Suramin, is an antagonist of P2X purinergic receptors -ion channels permeable to calcium that open upon binding of ATP- [159]. Delays in disease progression for patients with hormone-refractory prostate (HPRC) cancer treated with the P2X antagonist support the potential role of suramin as an anti-neoplasic therapy in PCa [160]. Another study of lung metastases induced by PCa cells showed reduction of tumor size, a decrease of non-apoptotic cells, and increased apoptotic cell number by suramin [161].

## 4. Conclusions

Dysregulation of calcium homeostasis plays an important role in PCa progression. Several mechanisms that increase or decrease [Ca2^+^]_i_ and a diversity of calcium-binding proteins regulate the various phases of PCa development. Different mechanisms allow PCa cells to mantain certain elevated levels of [Ca2^+^]_i_ that induce proliferation, angiogenesis, EMT, migration and bone colonization meanwhile other mechanisms guarantee evasion of [Ca2^+^]_i_ overload that could lead to mitochondria-dependent apoptosis. Future calcium-based therapies must specifically target prostate cancer cells either avoiding calcium entry or potentiating [Ca2^+^]_i_ overload that leads to apoptosis without affecting non-tumor cells.

## Figures and Tables

**Table 1:** Clinical trials regarding prostate cancer treatment using calcium-targeted therapies.

Treatment	Results	Recruitment Status	Phase	Interventions	Conditions	Clinicaltrials.Gov Identifier	Study Title:
							Study: Suramin (antagonist of P2X purinergic receptors)
Patients receive low, intermediate or high-dose suramin IV over 1 hour on days 1, 2, 8, 9, 29, 30, 36, 37, 57, 58, 64, and 65 in the absence of disease progression or unacceptable toxicity. Patients with new progression after partial or complete response may receive additional courses, at the discretion of the study chairperson.	No Study Results Posted on ClinicalTrials.gov for this Study	Completed	Randomized phase III trial to compare the effectiveness of low, intermediate, and high dose suramin	Low (3.192g/square meter total dose given decreasing concentrations in 250 cc normal saline IV), Intermediate (5.320 g/square meter total dose given in decreasing concentrations in 250 cc normal saline via IV), or High (7.661 g/square meter toal dose given in decreasing concentrations in 250 cc normal saline IV) Dose Suramin	Stage IV prostate cancer that is refractory to hormone therapy	NCT00002723	Low, Intermediate, or High Dose Suramin in Treating Patients With Hormone-Refractory Prostate Cancer
Within 3 days after randomization, all patients receive daily flutamide. On day 4, patients undergo orchiectomy or begin monthly LHRH analogue therapy with leuprolide or goserelin. Patients randomized to receive suramin begin a 12-week course 8-25 days after orchiectomy/LHRH therapy. Hydrocortisone replacement therapy begins concomitantly with suramin and continues for at least 3 months after the completion of suramin treatment or until disease progression intervenes.	No Study Results Posted on ClinicalTrials.gov for this Study	Completed	Randomized phase III trial to evaluate the effectiveness of treatment with flutamide and suramin with or without hydrocortisone	ORCHIECTOMY/LHRH ANALOG + FLUTAMIDE + SURAMIN + HYDROCORTISONE VS ORCHIECTOMY/LHRH ANALOG + FLUTAMIDE	Metastatic or recurrent prostate cancer	NCT00002881	Flutamide, Suramin, and Hydrocortisone in Treating Patients With Prostate Cancer
	No Study Results Posted on ClinicalTrials.gov for this Study	Completed	Phase II Trial	Combine androgen blockage (Leuprolide and Flutamide) with suramin	Metastatic prostate cancer	NCT00001266	A Phase II Trial of Leuprolide + Flutamide + Suramin in Untreated Poor Prognosis Prostate Carcinoma
	No Study Results Posted on ClinicalTrials.gov for this Study	Completed	Phase I Trial	Suramin followed by doxorubicin in patients with advanced solid tumors.	Histologic or cytologic confirmation of malignant solid tumor including, but not limited to: Breast cancer Prostate cancer Colon cancer Adrenocortical tumors	NCT00003038	Combination Chemotherapy With Suramin Plus Doxorubicin in Treating Patients With Advanced Solid Tumors
							Study: Mipsagargin (G-202) [thapsigargin-based prodrug] Inhibitor of ER calcium ATPase (SERCA)
G-202 administered by intravenous infusion over one hour on Days 1, 2 and 3 of a 28-day treatment cycle. The G-202 dose will be 40 mg/m2 on Day 1 and 66.8 mg/m2 on Days 2 and 3.		Withdrawn	Phase 2 Study	G-202 dose will be 40 mg/m2 on Day 1 and 66.8 mg/m2 on Days 2 and 3.	Patients With Chemotherapy-Naïve Metastatic Castrate-Resistant Prostate Cancer	NCT01734681	Phase 2 Study of G-202 in Patients With Chemotherapy-Naïve Metastatic Castrate-Resistant Prostate Cancer
G-202 administered by intravenous infusion on Days 1, 2 and 3 of each 28-day cycle for up to 3 cycles.	No Study Results Posted on ClinicalTrials.gov for this Study	Completed	Phase II clinical trial	G-202	Patients With Adenocarcinoma of the Prostate	NCT02381236	G-202 in the Neoadjuvant Setting Followed by Radical Prostatectomy in Patients With Prostate Cancer
G-202 administered by intravenous infusion over 1 hour on Days 1, 2 and 3 of each 28-day cycle.	No Study Results Posted on ClinicalTrials.gov for this Study	Completed	Dose-Escalation Phase 1 Study	G-202 on Days 1, 2 and 3 of each 28-day cycle.	Advanced Prostate Cancer	NCT01056029	Dose-Escalation Phase 1 Study of G-202 (Mipsagargin) in Patients With Advanced Solid Tumors
							Study: SOR-C13 (synthetic peptide inhibitor of TRPV6 developed from the C-terminal region of soricidin
Patients receive TRPV6 calcium channel inhibitor SOR-C13 IV over 2 hours on days 1, 2, 8, 9, 15, 16, 22, and 23. Cycles repeat every 28 days in the absence of disease progression or unacceptable toxicity.	No Study Results Posted on ClinicalTrials.gov for this Study	Recruiting	Phase I dose-escalation Study	SOR-C13 IV	Solid tumors that have spread to other places in the body (advanced) and does not respond to treatment. Stage III Prostate Cancer Stage IIIA Prostate Cancer Stage IIIB Prostate Cancer Stage IIIC Prostate Cancer Stage IV Prostate Cancer Stage IVA Prostate Cancer Stage IVB Prostate Cancer	NCT03784677	SOR-C13 in Treating Patients With Advanced Refractory Solid Tumors
Intravenous solution for infusion, potential dose range 1.375 mg/kg to 6.12 mg/kg, dosing frequency 2 cycles with a cycle consisting of infusions on days 1-3 and days 8-10 followed by a 11 day off period.	No Study Results Posted on ClinicalTrials.gov for this Study	Completed	Phase I, Open-label, Dose Escalation Study	SOR-C13 potential dose range 1.375 mg/kg to 6.12 mg/kg	Subjects with a histologic diagnosis of solid tumor cancers of epithelial origin	NCT01578564	Safety and Tolerability Study of SOR-C13 in Subjects With Advanced Cancers Commonly Known to Express the TRPV6 Channel
